# Pan-Cancer Analysis of Atrial-Fibrillation-Related Innate Immunity Gene *ANXA4*

**DOI:** 10.3389/fcvm.2021.713983

**Published:** 2021-09-03

**Authors:** Tao Yan, Shijie Zhu, Yu Shi, Changming Xie, Miao Zhu, Yangyang Zhang, Chunsheng Wang, Changfa Guo

**Affiliations:** ^1^Department of Cardiovascular Surgery, Zhongshan Hospital, Fudan University, Shanghai, China; ^2^Department of Cardiovascular Surgery, Shanghai East Hospital, Tongji University School of Medicine, Shanghai, China; ^3^Department of Cardiovascular, The Eighth Affiliated Hospital, Sun Yat-sen University, Shenzhen, China; ^4^Department of Cardiovascular Surgery, Shanghai Chest Hospital, Shanghai Jiao Tong University, Shanghai, China

**Keywords:** atrial fibrillation, cancer, immune infiltration, proteomics, TCGA

## Abstract

**Background:** Atrial fibrillation (AF) is the most common tachyarrhythmia around the world. Cancer is one of the main causes of death worldwide. A recent study demonstrated that cancer was associated with an increased incidence of AF. In the present study, we aimed to explore possible mechanisms and potential common therapeutic targets between AF and cancer.

**Methods:** Differentially expressed proteins between AF and sinus rhythm were identified utilizing proteomics analysis. Weighted gene correlation network analysis was applied to cluster proteins into different modules and investigate associations between modules and AF. Hub immune-related genes were selected via InnateDB database and verified using qRT-PCR. RNA sequencing and clinical data of 33 different cancer types were achieved from The Cancer Genome Atlas (TCGA). The correlations between *ANXA4* expression and the prognosis were calculated utilizing Cox regression analysis and Kaplan-Meier survival analysis. Spearman's rank correlation test was used to assess associations between *ANXA4* and immune infiltration and DNA methylation. Enrichment analysis was performed through gene ontology and gene set enrichment analysis (GSEA).

**Results:***ANXA4* was identified as hub immune-related gene between AF and sinus rhythm. Expression levels of *ANXA4* increased in diverse cancer types. Survival analysis suggested prognostic significance of *ANXA4* expression levels in various cancer types. Immune correlation analysis indicated that *ANXA4* expression levels were associated with tumor immune infiltration in most cancer types. *ANXA4* might influence the efficacy of immunotherapy via tumor burden and microsatellite instability. GSEA results indicated that high *ANXA4* expression groups were mainly enriched in peroxisome, bile acid biosynthesis, and p53 pathway.

**Conclusion:***ANXA4* was identified as a hub immune-related gene in AF, which has never been reported. Pan-cancer analysis indicated its potential as a novel clinical prognostic marker and therapeutic target in diverse cancer types. *ANXA4* might play crucial roles in AF and cancer, and targeted therapy for *ANXA4* might reduce the incidence of AF in cancer patients.

## Introduction

Atrial fibrillation (AF) is the most common tachyarrhythmia worldwide, which is a significant burden for both patients and public health (1). Thromboembolism is the primary hazard of AF, and the overall risk of stroke in patients with AF is five times more than that in the general population, affecting morbidity, mortality and quality of life (2). Although a variety of common risk factors have been confirmed to contribute to the development of AF, including age, sex, hypertension, obesity, and diabetes, (3) the exact pathological mechanism of AF is still unclear. Cancer, whose occurrence is a complex process with multiple risk factors, is one of the leading causes of death globally, which affects millions of people worldwide (4). A recent study based on a large population reported on the European Society of Cardiology (ESC) Congress 2020 demonstrated that cancer patients were associated with an increased incidence of AF (5). However, studies on associations between AF and cancer are still rare, which is worthy of further research to explore its possible mechanisms and potential common therapeutic targets.

Increased evidence suggests that immunity is essential both in AF and cancer. Expression levels of a number of immune-mediated serum inflammatory biomarkers such as CRP, IL-6, and TNF-α have been confirmed to increase in patients with AF (6, 7). Histological substrates of atrial biopsies in patients with AF demonstrated that immune inflammatory cell infiltration was widespread in atrial tissues (8). A recent study indicated that M1 macrophages might be involved in the development of AF (9). Tumor microenvironment is closely related to tumor growth, metastasis, and invasion. CD8^+^ T cells, activated by interacting with antigens on antigen presenting cells, are important in the anti-tumor immune response (10). CTLA4 and PD1, expressing on T cells which can attenuate T cells response, have been applied as therapeutic targets for immunotherapy in diverse cancer types in the clinic.

In the present study, we aimed to identify immune-related proteins in AF using proteomics and to explore its roles in various cancer types, hoping to provide novel insights into associations between cancer and AF and reveal a potential therapeutic target to decrease the incidence of AF in cancer patients.

## Materials and Methods

### Data Acquisition and Processing

Three public datasets, GSE31821, GSE41177, and GSE79768, including 11 healthy donors and 27 AF patients, were downloaded from the Gene Expression Omnibus (GEO) database to verify the expression level of *ANXA4* between sinus rhythm (SR) and AF groups. The SVA package in the R language was utilized to remove the batch effect. The gene expression of different cancer cell lines was downloaded from the Cancer Cell Line Encyclopedia (CCLE) database (11). Gene expression data of normal tissues were extracted from the Genotype-Tissue Expression (GTEx) database (12). The Cancer Genome Atlas (TCGA) was used to achieve RNA sequencing data and clinical data of 33 different cancer types. A total of 2,308 genes playing a role in the innate immune response were downloaded from the InnateDB, a database providing a manually-curated knowledge base of the genes involved in mammalian innate immunity (13).

### Patients Selection

Included in this study were ten patients with paroxysmal AF and ten with sinus rhythm (SR). All patients had complete clinical history and medical examinations, including electrocardiogram, echocardiography, coagulation function. Left atrial appendage (LAA) tissues of patients with AF and SR were collected during cardiac surgery. The use of heart tissues was in full compliance with the Declaration of Helsinki and approved by the Medical Ethics Committee of East Hospital, Tongji University. All subjects participating in this study had signed written informed consent before surgery.

### Protein Lysis and Quantification

As we described before, (14) LAA tissues were cut into large pieces with scissors and washed three times with PBS. After centrifugation and discarding the supernatant, precipitation was added with an appropriate amount of SDS lysis solution and steel balls, then shook and ground in a tissue grinder until tissues were wholly broken. Protein quantification was determined with the BCA assay.

### Filter-Aided Sample Preparation Enzymolysis of Protein

An appropriate amount of samples added 1M DTT solution to a final concentration of 100 mM was incubated at 56°C for 1 h. UA buffer (8 M Urea, 150 mM Tris-HCl pH8.5) was taken to remove low molecular weight impurities, including SDS. IAA (50 mM IAA in UA), 50 mM NH_4_HCO_3_ solution, Trypsin buffer (5 μg Trypsin in 40 μL 50 mM NH_4_HCO_3_ solution) were also used for FASP enzymolysis of proteins. After the final filtrate was lyophilized, 50 μl of 0.1% TFA was added for dissolution. The peptides were desalted by the RP-C18 solid-phase extraction column through equilibrium (rinsed once respectively by 1 mL of methanol, 1 mL of 90% acetonitrile-water solution, and 1 mL of double-distilled water), adsorption (fully dissolved by 1 mL of double distilled water and naturally adsorbed three times by gravity), washing (rinsed by 0.1% trifluoroacetic acid-water solution three times), elution (eluted by 90% acetonitrile-water solution for three times) and redissolution (redissolved by 0.1% formic acid-water solution after vacuum drying).

### LC-MS/MS Analysis of Enzymolysis Products

According to the quantitative results, 1 μg of the enzymatic hydrolysis products were taken for LC-MS/MS analysis. The separation was carried out by a nanoliter flow rate HPLC liquid phase system EASY-nLC1000. A 0.1% formic acid-water solution was used for liquid phase A, and a 0.1% formic acid-acetonitrile solution for liquid phase B. The sample was loaded by the autosampler and adsorbed on the Trap column, then separated by the Analysis column (column temperature: 50°C, flow rate: 300 nl/min). The relevant liquid phase gradients are as follows: 0–6 min, the linear gradient of liquid B from 1 to 5%; 6–86 min, the linear gradient of liquid B from 5 to 26%; 86–106 min, the linear gradient of liquid B is from 26 to 40%; 106-111 min, the linear gradient of liquid B is from 40 to 100%; 111–116 min, the liquid B is maintained at 100%; 116–117 min, the linear gradient of liquid B from 100 to 1%. Separated products were analyzed by the Thermo Fisher fusion mass spectrometer. The profile mode was used for the first mass spectrometry and centroid mode for the second mass spectrometry to reduce the size of the data file.

### Database Search

Thirty LC-MS/MS original files were imported into MaxQuant software (version 1.6.0.1) for database search. Label-free quantification (LFQ) analysis was conducted through the search engine Andromeda. The database was downloaded from the UniProt database. The anti-database of UniProt Homo sapiens was used to calculate the false positive rate (FDR) of peptides and proteins. MaxQuant software integrated the LFQ algorithm by extracting the isotope peaks of each peptide in each analysis. The platform calculated the protein ratio using the median of the ratio of common peptides in all analyses, which represents a relatively approximate estimate of the protein ratio. The data obtained from MaxQuant analysis was imported into Perseus (version 1.5.1.6) software for further investigation to filter out the proteins identified only by site, reverse database, and common contaminant database.

### Weighted Gene Correlation Network Analysis (WGCNA)

Wgcna is a system biology method utilized to cluster genes or proteins into modules based on the interconnectivity, which can calculate correlations between modules and clinical phenotypes to identify hub genes (15). The WGCNA was utilized to construct the co-expression network based on the proteins identified using proteomics in this study. First, an appropriate soft-thresholding power β was calculated to realize the scale-free topology with the criterion of *R*^2^ > 0.85. Then the average linkage hierarchical clustering method was used to cluster proteins into different modules labeled with different colors. Each module contained at least twenty proteins, and the threshold was set as 0.25 for module merging. Pearson's correlation method was applied to determine the correlation between each module and AF. The module with a *p*-value < 0.05 and the highest correlation coefficient was selected for further analysis.

### Functional Enrichment Analysis

Gene Ontology (GO) enrichment analyses of proteins were carried out using the Database for Annotation, Visualization and Integrated Discovery (DAVID) (16) GO terms with a *p*-value < 0.05 were considered significant enrichment. Metascape, a website developing reliable, productive and intuitive tools to analyze gene or protein lists, was also used for enrichment analysis (17). GeneMANIA, an online tool including association data of protein and genetic interactions, pathways, co-expression, co-localization, and protein domain similarity, was applied to construct the protein-protein interaction (PPI) network (18). Gene set enrichment analysis (GSEA) was performed to investigate biological signaling pathway between the high *ANXA4* expression group and the low *ANXA4* expression group according to the median expression level of *ANXA4* (19).

### Quantitative Real-Time PCR (qRT-PCR)

Total cellular RNA from LAA tissues was extracted using the RNeasy^TM^ Mini Kit (QIAGEN, Frankfurt, Germany), following the manufacturer's instructions. The Complementary DNA (cDNA) was synthesized by reverse transcription at 42°C for 60 min and then at 95°C for 5 min with the PrimeScript^TM^ RT reagent Kit (Takara, Otsu, Japan). TB Green^®^ Premix Ex Taq^TM^ II (Takara, Otsu, Japan) was applied to perform qRT-PCR at the temperature of 95°C for 30 seconds, followed by 40 cycles with the temperature of 95°C for 5 seconds and 60°C for 34 seconds on QuantStudio^TM^ 5 System (Thermo Fisher Scientific, Waltham, MA, USA). The expression of RNA levels was normalized by GAPDH, and the 2^−ΔΔCT^ method was applied to calculate the relative expression with three independent repeats. All sequences for RNA primers (Sangon Biotech, Shanghai, China) are shown in Table 1.

**Table 1:** Lists of primer sequences used for quantitative real-time PCR.

**Genes**	**Sequences**
*GAPDH*	Forward: GGAGCGAGATCCCTCCAAAAT
	Reverse: GGCTGTTGTCATACTTCTCATGG
*ANXA4*	Forward: GGAGGTACTGTCAAAGCTGCT
	Reverse: GGCAAGGACGCTAATAATGGC

### Survival Analysis

Kaplan-Meier survival analysis was performed to calculate the associations between the prognosis of patients, including overall survival (OS), disease-specific survival (DSS), and progression-free interval (PFI) and expression levels of *ANXA4* in 33 different cancer types. Hazard ratio (HR) with 95% confidence intervals and *p*-value were determined using univariate Cox regression analysis and Log-rank test, respectively.

### Immune Infiltrating Analysis

The Tumor Immune Evaluation Resource (TIMER) database is a comprehensive resource for systematically analyzing immune infiltrates across diverse cancer types estimated by multiple immune deconvolution methods (20). Spearman correlation analysis was used to estimate the associations between the expression levels of *ANXA4* and infiltrating immune scores of B cells, CD4^+^ T cells, CD8^+^ T cells, dendritic cells, macrophages, and neutrophils. The xCell is a webtool based on gene signatures that performs cell type enrichment analysis from gene expression data for immune cell types (21). The correlations between *ANXA4* expression levels and different immune cell types were calculated utilizing xCell. Estimation of Stromal and Immune Cells in Malignant Tumor Tissues Using Expression Data (ESTIMATE) was used to predict the purity of a tumor based on infiltration of stromal cells and immunocytes, which was shown in the form of three different scores, including stromal score, immune score, and estimate score (22). The correlation between *ANXA4* expression and these three kinds of scores was calculated using the ESTIMATE algorithm. We also examined correlations between common immune checkpoints and *ANXA4* expression levels using Spearman's rank correlation test.

### Mutation and DNA Methylation

Tumor mutational burden (TMB), which refers to the number of somatic mutations per megabase in the coding region, is a new genetic biomarker predicting the efficacy of immunotherapy. The correlation between the *ANXA4* expression level and TMB was analyzed using Spearman's rank correlation test. Microsatellite instability (MSI) is defined as any change in the length of a microsatellite during DNA replication, resulting in the emergence of new microsatellite alleles. Spearman's rank correlation test was performed to calculate the association between *ANXA4* levels and MSI. Mismatch repair (MMR) is a DNA repair mechanism, restoring the nucleotide sequence to normal in DNA molecules containing mismatched bases. We also analyzed the correlation between MMR genes (*MLH1, MSH2, MSH6, PMS2*, and *EPCAM*) and the expression level of *ANXA4*. DNA methylation is a form of chemical modification that can change genetic performance without changing the sequence. Under the action of methylase, DNA methylation cause changes in chromatin structure, DNA conformation, DNA stability, and the way of interaction between DNA and protein, thereby controlling gene expression. Relationships between the expression of four DNA methyltransferases (DNMT1, DNMT2, DNMT3A, and DNMT3B) and *ANXA4* expression levels in different cancer types were determined.

## Results

### Baseline Characteristics of Patients

A total of ten male paroxysmal NVAF patients were included in the study, with a mean age of 64.9 ± 3.0 years (range from 61 to 69 years). All patients underwent echocardiography and coronary angiography to rule out valve diseases and coronary heart disease. Detailed baseline characteristics are shown in Table 2.

**Table 2:** Baseline characteristics of patients.

**Parameters**	**Values**
Gender, *n* (%)	
Male	10 (100%)
Female	0 (0)
Age, yr, mean±SD, (range)	64.9 ± 3.0 (61 to 69)
LA diameter, mm, mean±SD, (range)	40.7 ± 3.2 (36 to 46)
LVEF, %, mean±SD, (range)	64.4 ± 3.6 (59 to 70)
BMI, kg/m^2^, mean±SD, (range)	24.6 ± 3.5 (18.4 to 31.2)
Medical history, *n* (%)	
Hypertension	6 (60%)
Diabetic mellitus	2 (20%)
History of preoperative stroke	0 (0)
History of catheter ablation	0 (0)
Valve diseases	0 (0)
Coronary heart disease	0 (0)

*LA, left atrium; LVEF, left ventricular ejection fraction; BMI, body mass index*.

### Proteins Identification

Differentially expressed proteins (DEPs) between paroxysmal NVAF and SR were identified utilizing LC-MS/MS and LFQ proteomics. A total of 1983 DEPs and 23113 unique peptides were identified for subsequent analysis.

### WGCNA

**Figure 1:**
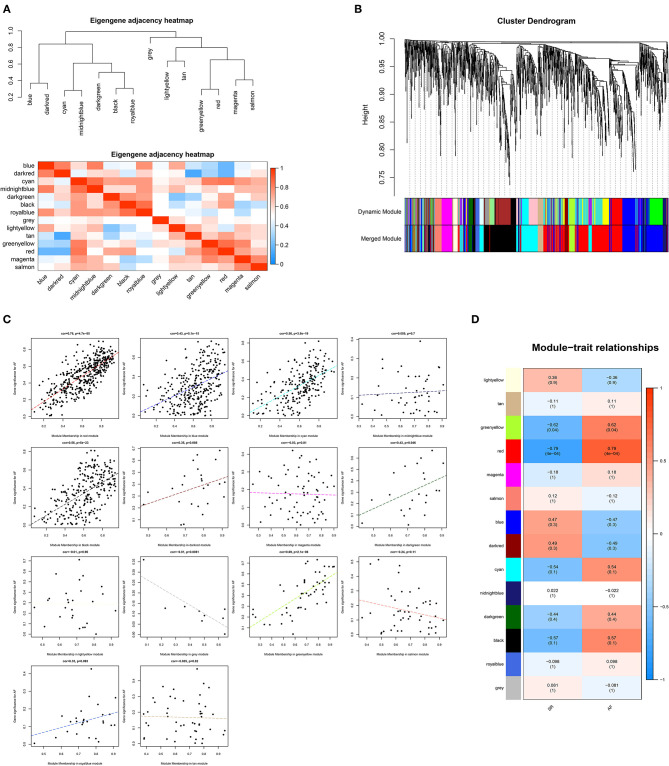
Weighted genes correlation network analysis. **(A)** The clustering heatmap between modules. Red means closer similarity and blue means farther similarity. **(B)** The dendrogram of differentially expressed genes. **(C)** The correlation between module memberships and the gene significance for atrial fibrillation. The abscissa represents the module membership in each module. **(D)** The heatmap of module-trait correlations. Blue represents negative correlation, and red represents positive correlation.

Based on the proteins identified above, a total of 1983 DEPs were subjected to WGCNA. A scale-free (scale-free *R*^2^ > 0.85) coexpression network with the soft-thresholding power β = 7 was then established. The average linkage hierarchical clustering method was applied to cluster DEPs into 14 modules with different colors, including blue, dark-red, cyan, midnight-blue, dark-green, black, royal-blue, light-yellow, tan, green-yellow, red, magenta, salmon, and gray. Genes in the gray module were excluded from further analysis due to the lack of correlation (Figure 1). Correlation analysis was performed between each module and AF, showing that the red module with 419 proteins was positively correlated with AF with the highest correlation coefficient (*r* = 0.79, *p* < 0.001).

### Enrichment Analysis

**Figure 2:**
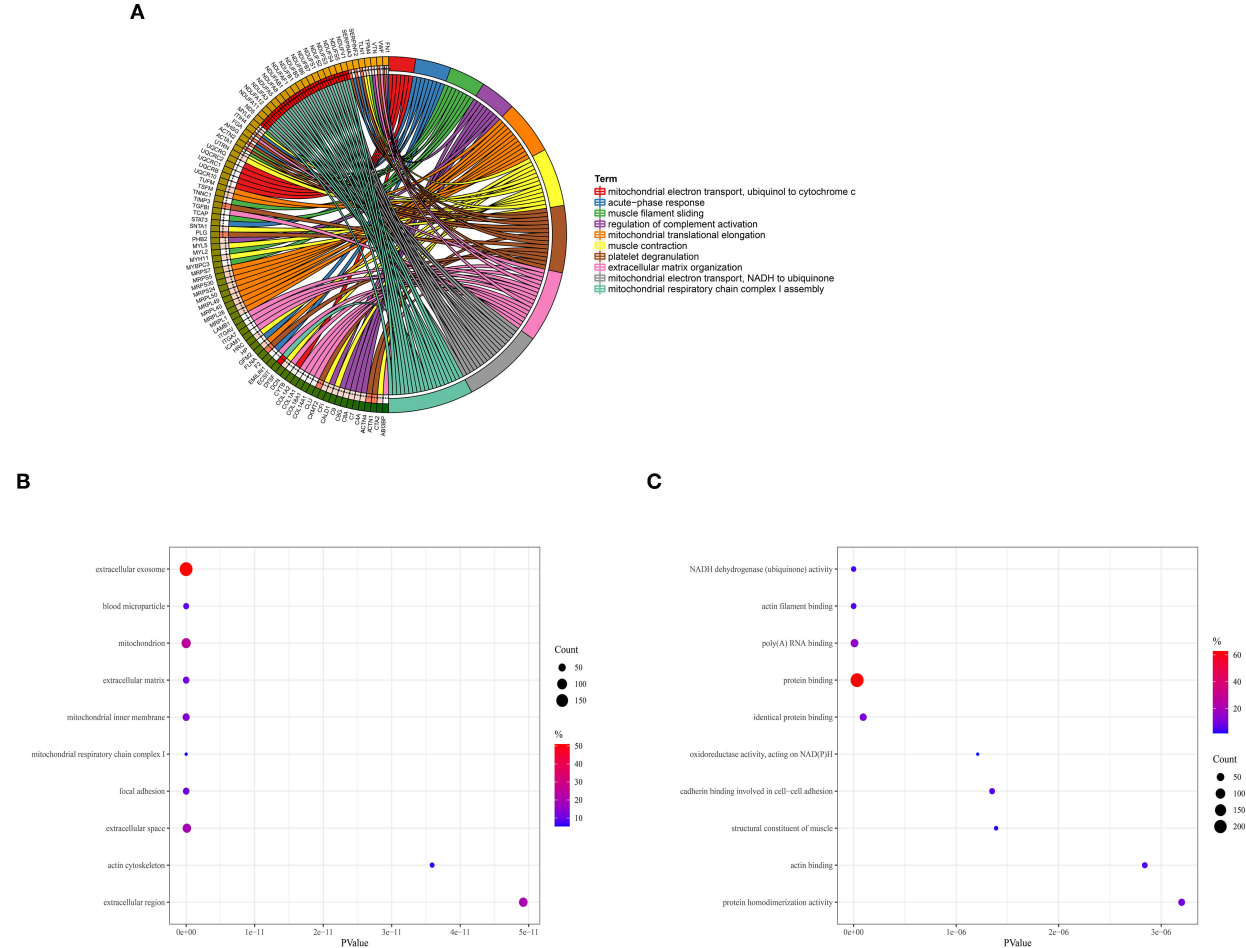
Gene Ontology enrichment analysis. **(A)** Biological process. **(B)** Cellular component. **(C)** Molecular function.

DAVID online tool was utilized to perform GO functional enrichment analysis on proteins in the red module to investigate the biological effects. The significant enriched biological processes included mitochondrial electron transport from NADH to ubiquinone, mitochondrial respiratory chain complex I assembly, platelet degranulation, mitochondrial translational elongation, and muscle contraction (Figure 2A). For cellular components, the most significant entries were extracellular exosome, blood microparticle, mitochondrion, extracellular matrix, and mitochondrial inner membrane (Figure 2B). In addition, NADH dehydrogenase activity, actin filament binding, poly-A RNA binding, protein binding, and identical protein binding were significantly enriched in molecular function (Figure 2C).

### Hub Immune-Related Genes Identification and Validation

**Figure 3:**
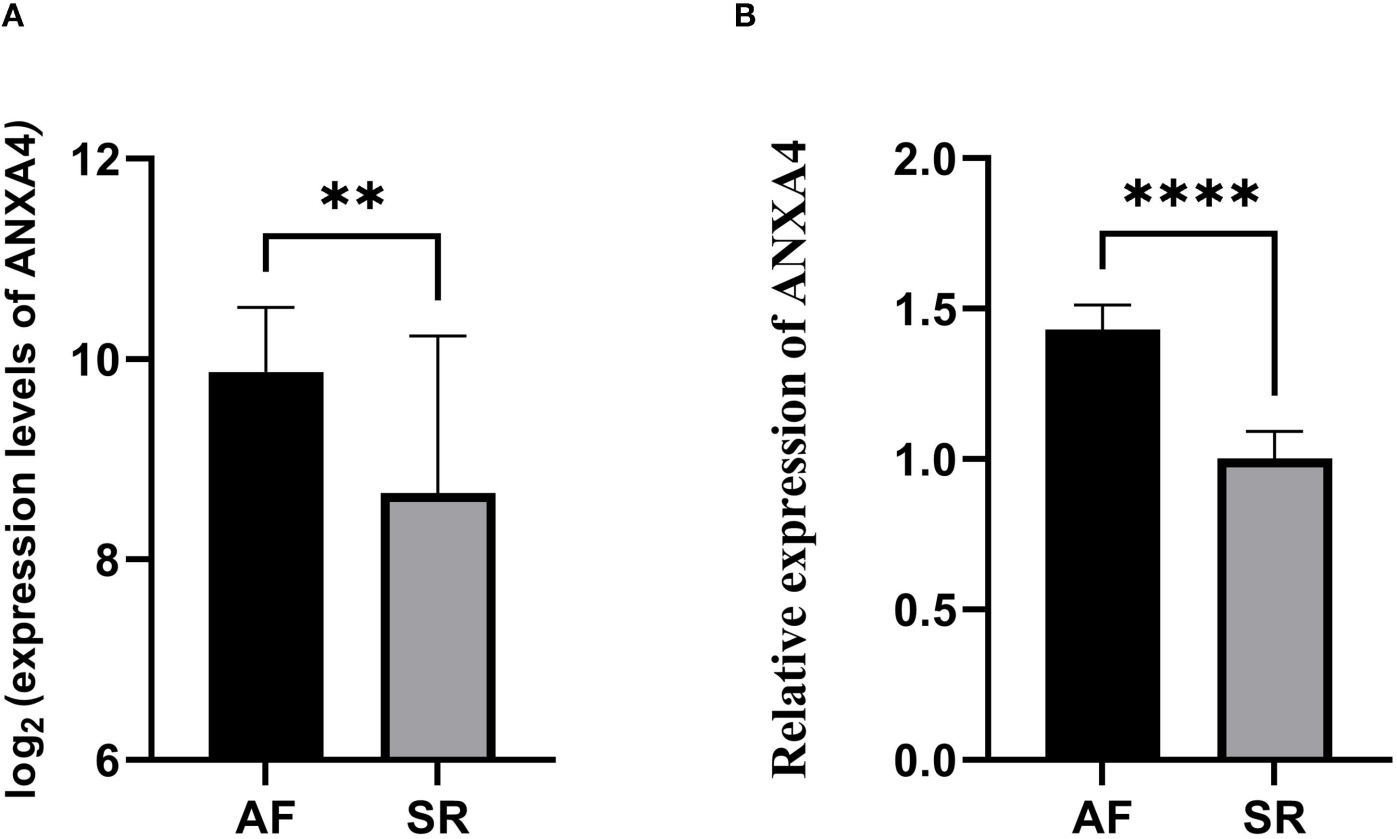
The relative expression of *ANXA4* between atrial fibrillation and sinus rhythm. **(A)** The relative expression of *ANXA4* verified by three datasets. **(B)** The relative expression of *ANXA4* in left atrial appendage tissues. **p < 0.01, ****p < 0.0001.

Considering the essential roles of immune response in AF and cancer, we downloaded 2,308 immune-related genes playing roles in the immune response from the InnateDB. According to eigengene connectivity through WGCNA, a total of 13 hub genes were screened out with eigengene connectivity more than 0.9 in the red module. Taking the intersection between 2,308 immune-related genes and 13 hub genes, *ANXA4* was identified as an immune-related gene that might regulate the pathogenesis of AF. We then performed qRT-PCR to verify the expression level of *ANXA4* in AF. The results demonstrated that the *ANXA4* expression level in AF was significantly higher than that in SR, which was consistent with the proteomic analysis (Figure 3).

### The Expression of ANXA4 in Different Cancer Types

**Figure 4:**
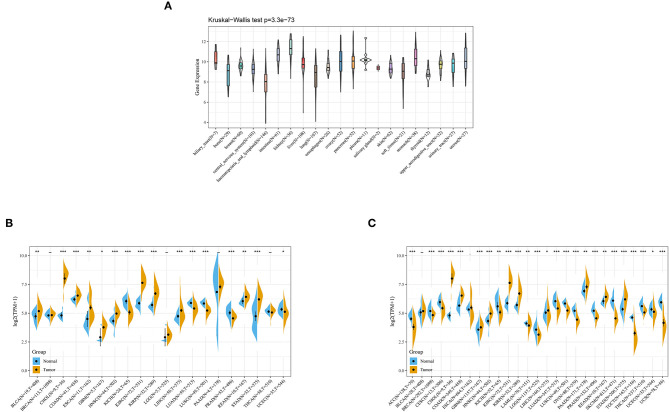
*ANXA4* expression in different cancer types. **(A)** Expression levels of *ANXA4* in different cancer cell lines. **(B)** Expression levels of *ANXA4* in TCGA. **(C)** Expression levels of *ANXA4* in combination with the GTEx database and TCGA. *p < 0.05, **p < 0.01, ***p < 0.001.

Data of diverse tumor cell lines were downloaded from the CCLE database. Expression levels of *ANXA4* in different cell lines were calculated, and as shown in Figure 4A, *ANXA4* expressed stably in 21 tumor cell lines. Then *ANXA4* expression levels between tumor and normal tissues were analyzed using data from the TCGA database. The results demonstrated that expression levels of *ANXA4* were significantly increased in most cancer types, including BLCA, CHOL, COAD, ESCA, GBM, HNSC, KIRC, KIRP, LIHC, READ, and STAD (Figure 4B). We then combined with the GTEx database to analyze the expression differences of *ANXA4* between tumor tissues and normal tissues in 27 different cancer types due to small numbers of normal tissues in TCGA. The results suggested that *ANXA4* expression levels were higher in CHOL, COAD, GBM, HNSC, KIRC, KIRP, LIHC, PAAD, READ, and STAD, indicating *ANXA4* might act as an oncogene in many cancer types (Figure 4C).

### Survival Analysis

**Figure 5:**
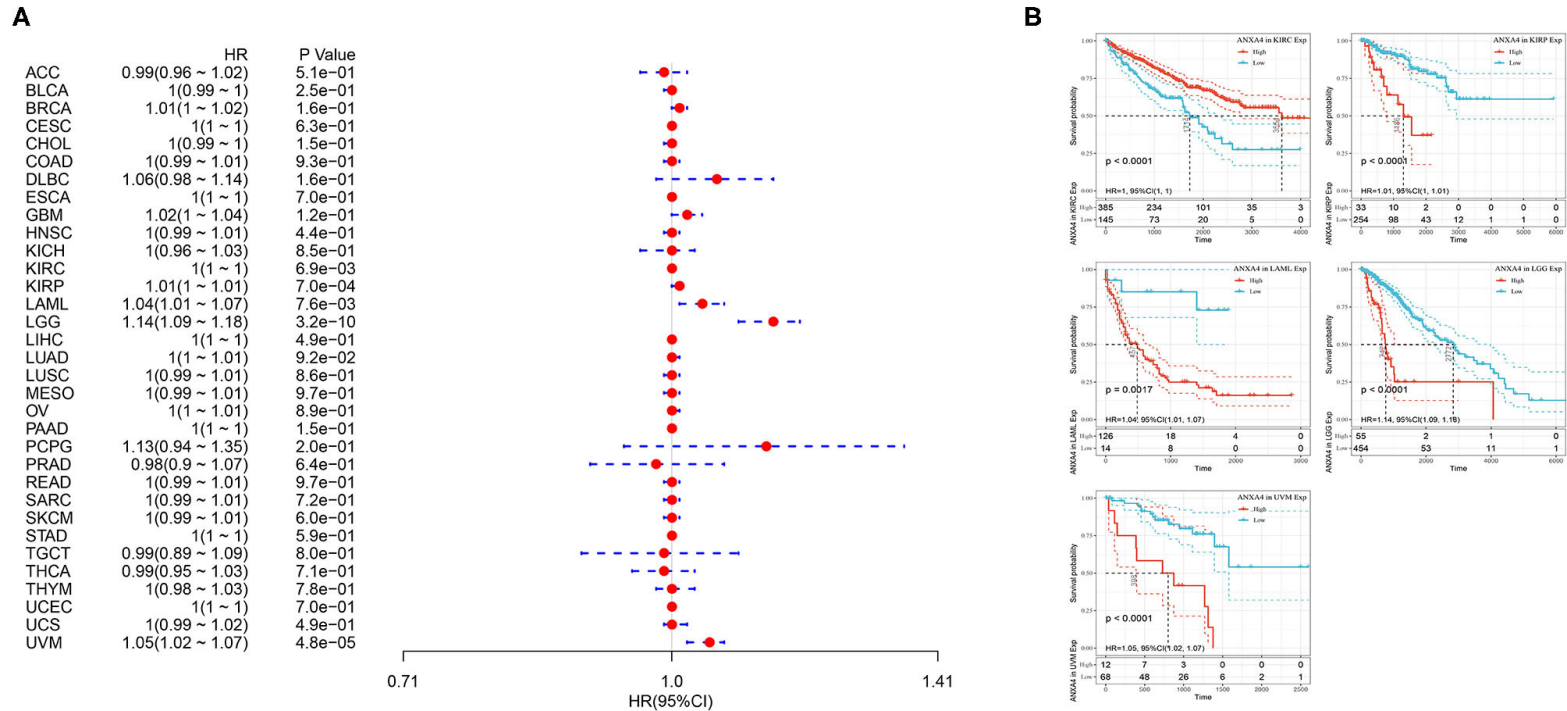
Relationships between *ANXA4* expression levels and prognosis. **(A)** Forest plots of overall survival (OS). **(B)** Kaplan-Meier curves for OS.

The associations between OS of patients and *ANXA4* expression levels were analyzed using Kaplan-Meier survival analysis. The results indicated that increased *ANXA4* expression levels negatively correlated with OS significantly in KIRP (*p* < 0.0001), LAML (*p* = 0.0017), LGG (*p* < 0.0001), and UVM (*p* < 0.0001), while positively correlated with OS in KIRC (*p* < 0.0001) (Figure 5). Furthermore, high expression levels of *ANXA4* were found to result in low DSS in patients with KIRP (*p* < 0.0001), LGG (*p* < 0.0001), and UVM (*p* < 0.0001), while result in high DSS in patients with KIRC (*p* < 0.0001) (Supplementary Figures 1A,C). Moreover, the correlations between PFI and *ANXA4* expression levels were also calculated, and we found that higher *ANXA4* expression was associated with lower PFI in KIRP (*p* < 0.0001), LGG (*p* < 0.0001), and UVM (*p* = 0.00042) (Supplementary Figures 1B,D). Results of survival analysis suggested prognostic significance of *ANXA4* expression levels in diverse cancer types.

### Immune Infiltration

**Figure 6:**
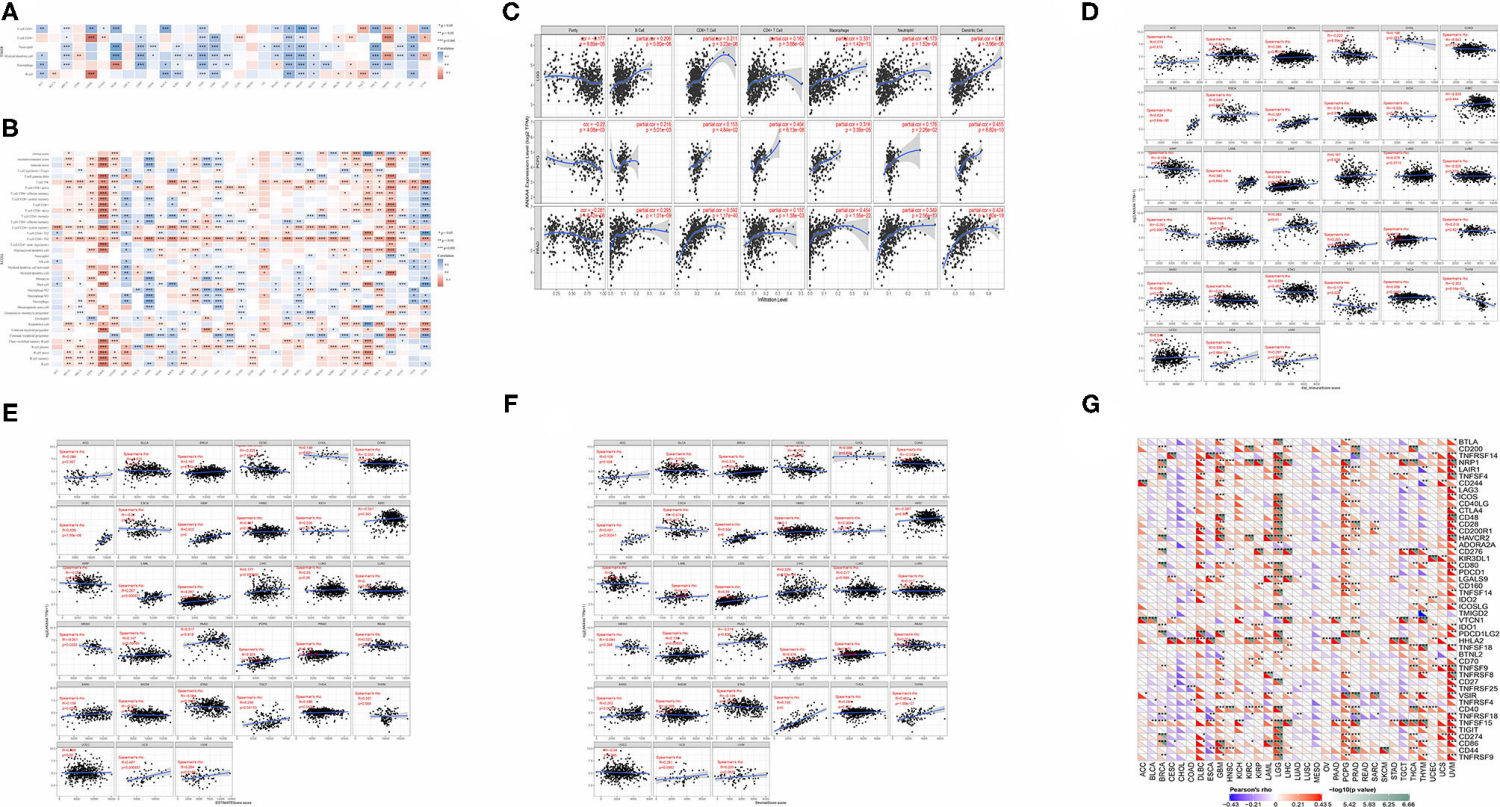
Correlations between *ANXA4* expression and immune infiltration. **(A)** Correlations between *ANXA4* expression levels and immune infiltration in the TIMER database. **(B)** Correlations between *ANXA4* expression levels and immune infiltration utilizing xCell. **(C)** Relationships between *ANXA4* expression levels and immune infiltrate in top three relevant tumor types. **(D)** Immune score calculated through ESITIMATE. **(E)** Estimate score calculated through ESITIMATE. **(F)** Stromal score calculated through ESITIMATE. **(G)** Correlations between *ANXA4* expression levels and immune checkpoints markers. *p < 0.05, **p < 0.01, ***p < 0.001.

To determine the role of *ANXA4* in tumor infiltrating lymphocytes abundance, the TIMER database was applied to estimate correlations between *ANXA4* expression and infiltration levels of different immune cells (Figures 6A,C). The results indicated that *ANXA4* expression was significantly associated with B cell in 15 cancer types, macrophage in 17 cancer types, dendritic cell in 20 cancer types, neutrophil in 16 cancer types, CD4^+^ T cell in 14 cancer types, and CD8^+^ T cell in 16 cancer types. The expression levels of *ANXA4* were markedly positively correlated with infiltrating immune cells in most cancer types, especially in LGG, LIHC, PCPG, PRAD, and THCA. Only in ESCA and MESO, *ANXA4* levels suggested no significant correlation with any immune cells. Further analysis utilizing the xCell online tool also demonstrated the association between *ANXA4* levels and infiltrating immune cells (Figure 6B). Higher *ANXA4* expression significantly decreased CD4^+^ Th1 cells across 30 cancer types. ESTIMATE algorithm was used to calculate the stromal score, immune score, and estimate score of infiltration of stromal cells and immunocytes (Figures 6D–F). Cancer types significantly correlated with these three scores included BRCA, CESC, DLBC, GBM, LGG, LIHC, OV, PCPG, PRAD, TGCT, and UCS. The most three tumor types relevant to *ANXA4* expression levels were DLBC, GBM, and UCS (immune score), DLBC, GBM, and UCS (estimate score), TGCT, GBM, and THYM (stromal score). Immune checkpoints were essential in immune response and immunotherapy. Correlations between expression levels of *ANXA4* and common immune checkpoints were also examined (Figure 6G).

### TMB, MSI, MMR, and DNA Methylation

**Figure 7:**
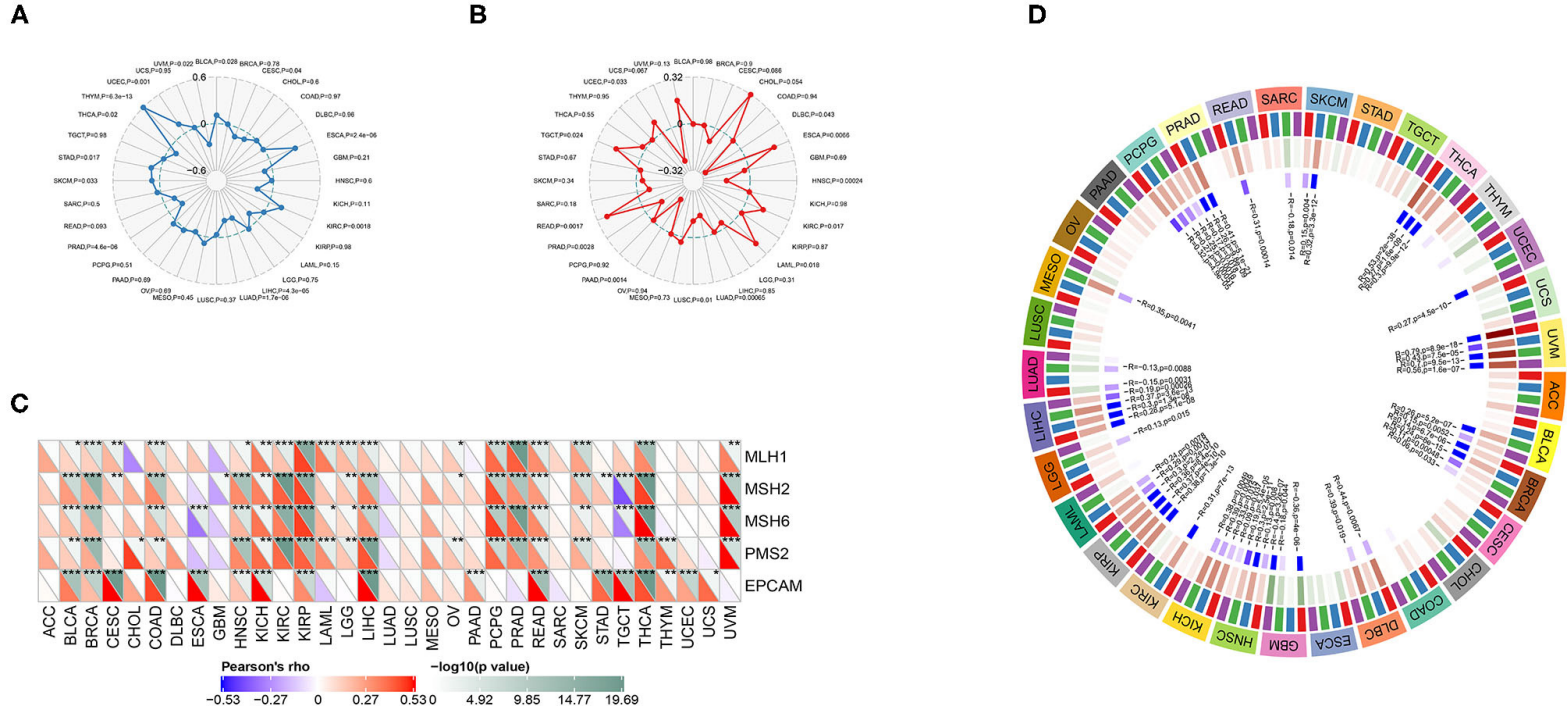
Correlations between *ANXA4* expression levels and tumor mutational burden (TMB), microsatellite instability (MSI), mismatch repair (MMR), and DNA methylation. **(A)** The Rader chart showing correlation between expression levels of *ANXA4* and TMB. **(B)** The Rader chart showing correlation between expression levels of *ANXA4* and MSI. **(C)** Correlations between *ANXA4* expression levels and MMR genes (*MLH1, MSH2, MSH6, PMS2*, and *EPCAM*). *p < 0.05, **p < 0.01, ***p < 0.001 **(D)** Correlations between *ANXA4* expression levels and DNA methyltransferases (red: DNMT1, blue: DNMT2, green: DNMT3A, purple: DNMT3B).

*ANXA4* was significantly correlated with TMB in BLCA, CESC, ESCA, KIRC, LIHC, LUAD, PRAD, SKCM, STAD, THCA, THYM, UCEC, and UVM (Figure 7A). Higher expression of *ANXA4* was correlated with higher TMB in BLCA, ESCA, KIRC, SKCM, STAD, THYM, and UCEC, indicating better OS and the potential of immunotherapy. The associations between *ANXA4* expression levels and MSI were significant in DLBC, ESCA, HNSC, KIRC, LAML, LUAD, LUSC, PAAD, PRAD, READ, TGCT, and UCEC (Figure 7B). We then calculated the correlations between *ANXA4* and MMR genes, including *MLH1, MSH2, MSH6, PMS2*, and *EPCAM*. As shown in Figure 7C, *ANXA4* was associated with all five MMR genes in nine cancer types, including BLCA, BRCA, COAD, HNSC, KICH, KIRP, LIHC, READ, and THCA. These results indicated that *ANXA4* might affect immunotherapy in various cancer types via TMB, MSI, or MMR. We next examined correlations between *ANXA4* levels and DNA methyltransferases, including DNMT1, DNMT2, DNMT3A, and DNMT3B (Figure 7D). We found that expression levels of *ANXA4* were significantly correlated with DNA methylation in diverse cancer types, especially in BRCA, HNSC, KIRP, LIHC, PCPG, and UVM.

### PPI Network and GSEA

**Figure 8:**
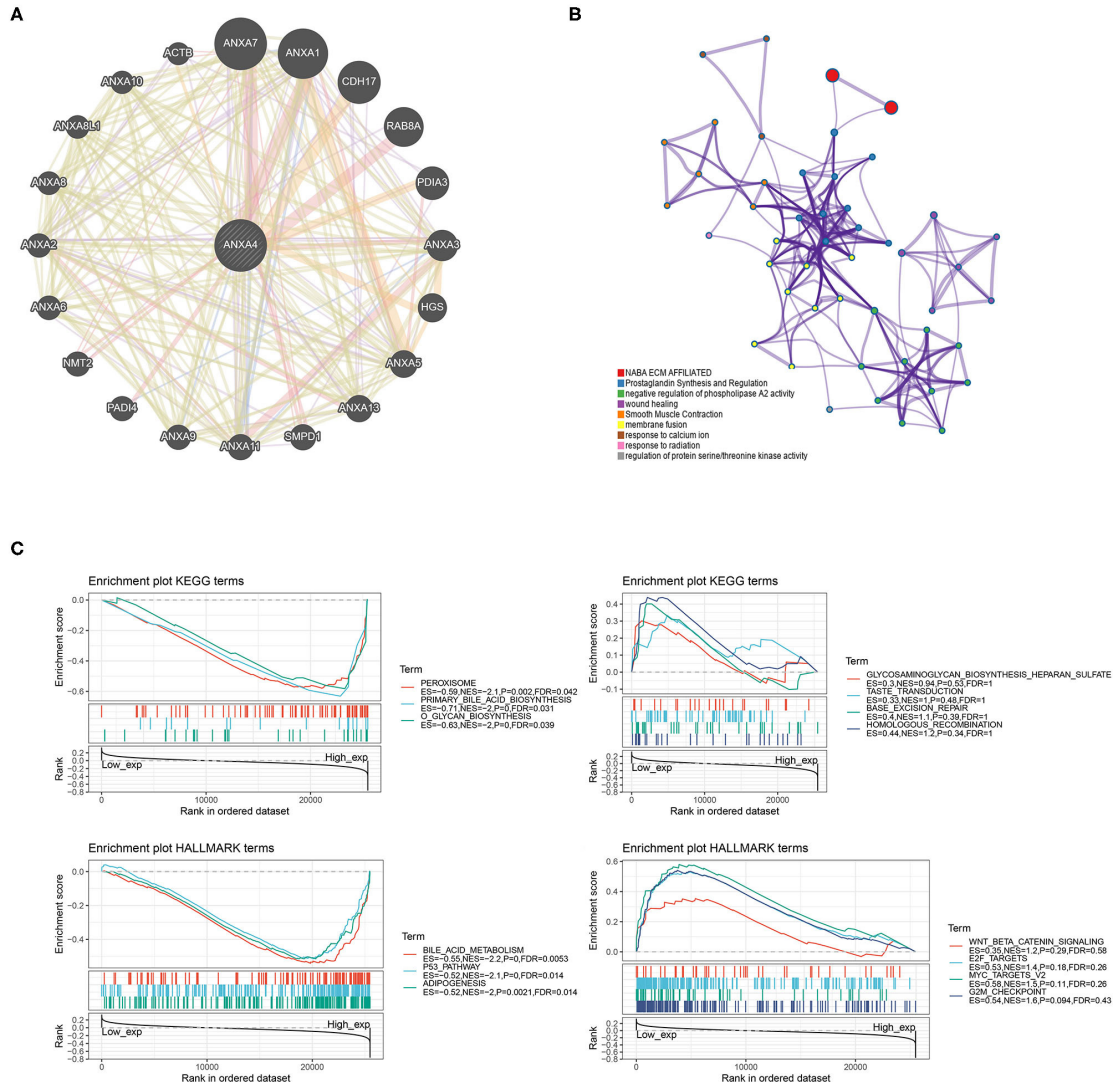
Protein-protein interaction (PPI) network and enrichment analysis (GSEA). **(A)** Construction of a PPI network with 21 genes centered on *ANXA4*. **(B)** Enrichment analysis using Matescape. **(C)** Gene set enrichment analysis (GSEA).

A PPI network with 21 genes centered on *ANXA4* was constructed using GeneMANIA (Figure 8A). The Metascape was utilized to perform enrichment analysis on these 21 genes (Figure 8B). The significantly enriched entries included prostaglandin synthesis and regulation, negative regulation of phospholipase A2 activity, response to calcium ion, response to radiation, and regulation of protein serine/threonine kinase activity. GSEA results demonstrated that KEGG terms in the high *ANXA4* expression group were mainly enriched in peroxisome, primary bile acid biosynthesis, and O-glycan biosynthesis. Hallmark enrichment terms suggested that high *ANXA4* expression was significantly associated with bile acid metabolism, p53 pathway, and adipogenesis (Figure 8C).

## Discussion

AF is one of the leading causes of stroke, leading to morbidity and mortality, affecting patients' quality of life (23). Although a considerable number of studies on AF have been studied from bench to bedside, the pathophysiological mechanism of AF is still unclear. The occurrence and development of AF are related to various risk factors, such as age, sex, genetics, and obesity. Moreover, other common diseases are often associated with AF, including hypertension, diabetes, and valve diseases (24, 25). A recent large-population study exhibited on the ESC Congress 2020 suggested that cancer patients were associated with an increased incidence of AF. However, research on the mechanism between AF and cancer has hardly been reported.

In the present study, 20 LAA tissues underwent proteomics analysis, and a total of 1,983 DEPs were identified between AF and SR groups. WGCNA was then utilized to explore modules related to AF, and the red module with the highest correlation coefficient was selected for further analysis. GO enrichment analysis demonstrated that the significantly enriched biological processes included mitochondrial electron transport from NADH to ubiquinone, mitochondrial respiratory chain complex I assembly, and mitochondrial translational elongation. Mitochondria, whose function is regulated by Ca^2+^, are the primary producers of cellular ATP in cardiac myocytes (26, 27). A recent study focused on electron microscopic features of LAA tissues demonstrated mitochondrial aggregates and an increase in mitochondria in patients with AF (28). Increased evidence suggested that mitochondrial function is impaired in patients with AF (29). Decreased expression levels of diverse enzymes contributing to mitochondrial energy metabolism were observed in AF (30). Mitochondria is also one of the main enzymatic systems producing reactive oxygen species which participate in cardiac fibrosis in AF (31, 32). Moreover, mitochondria play central roles in cancer. Mitochondrial ubiquinol oxidation is necessary for tumor growth (33). Another study suggested that mitochondrial complex III is essential for the suppressive function of regulatory T cells (34). Mitochondria is applied as critical therapeutic targets because of its central role in cellular proliferation and death (35). Impaired mitochondrial function may be a common mechanism of AF and cancer, contributing to increased incidence of AF in cancer patients.

Considering the essential roles of immune response in AF and cancer, an intersection between 2308 immune-related genes downloaded from the InnateDB and 13 hub genes screened out with eigengene connectivity more than 0.9 in the red module was taken. *ANXA4* was identified as the hub immune-related gene. *ANXA4* is a protein-coding gene that belongs to the annexin family of calcium-dependent phospholipid-binding proteins. *ANXA4* N-terminal peptide inhibits adenylyl cyclase 5 and limits beta-adrenoceptor-mediated prolongation of the cardiac action potential (36). However, *ANXA4* has never been reported in AF, which deserves further research.

We then analyzed *ANXA4* expression levels and found increased *ANXA4* expression in diverse cancer types, indicating that *ANXA4* might act as an oncogene. Survival analysis showed prognostic significance of *ANXA4*. Targeted therapy for *ANXA4* might relieve tumors and improved the prognosis of patients in different cancer types. Few previous studies were found between *ANXA4* and cancer. In ovarian carcinoma, p53 and *ANXA4*/NF-κB p50 complexes regulate cell proliferation, apoptosis, and tumor progression (37). The overexpression of Lewis(y), a component of the structure of the *ANXA4* membrane protein, induces the chemoresistance of ovarian cancer cells, and ultimately promoting the progression of ovarian cancer (38). It was reported that the increased serum level of *ANXA4* might be a promising biomarker for the early detection of hepatocellular carcinoma (39). A recent study indicated that downregulation of *ANXA4* by toosendanin could mediate cisplatin sensitization in non-small cell lung cancer (40).

Furthermore, the correlations between immune infiltration and *ANXA4* expression levels in various cancer types were calculated. TIMER and xCell suggested that *ANXA4* were markedly positively correlated with tumor infiltrating lymphocytes in most cancer types, affecting tumor development via tumor microenvironment (41–43). CTLA-4 and PD-1 inhibitors are immune checkpoint inhibitors showing acceptable results in lung cancer and melanoma. We collected 40 common immune checkpoints and examined the associations between *ANXA4* expression levels and immune checkpoints to find its potential as a further therapeutic target in the clinic. Moreover, a 21-genes PPI network centered on *ANXA4* was constructed. The enrichment analysis demonstrated that these genes were enriched in prostaglandin synthesis and regulation, negative regulation of phospholipase A2 activity, response to calcium ion, and response to radiation. Prostaglandin is related to inflammation, which plays a crucial role in both AF and cancer (44–46). GSEA results indicated that the high *ANXA4* expression group was mainly enriched in peroxisome, bile acid, and p53 pathway, which were all associated with different cancer types.

Although the integrative analysis on *ANXA4* was performed using different databases and algorithms, there are some limitations in the present study. First, *ANXA4* has never been reported in AF. The further experiment should be performed to explore and verify its potential mechanism in AF. Second, *ANXA4* was identified through proteomics analysis and verified using qRT-PCR. However, the roles of *ANXA4* in cancers were analyzed solely based on our bioinformatics analysis, whereas *in vitro* or *in vivo* experiments should be conducted to elucidate the molecular mechanisms of *ANXA4* in different cancer types. Third, further prospective studies on cancer patients with AF should be carried out to evaluate the efficacy of *ANXA4* in reducing the incidence of AF in cancer patients.

## Conclusion

In short, the present study identified *ANXA4* as a hub immune-related gene, which has never been reported in AF. The pan-cancer analysis suggested that *ANXA4* was significantly associated with prognosis and immune infiltration in various cancer types, which has the potential as a novel biomarker in cancer patients. Considering its role in AF and cancer, targeted therapy for *ANXA4* might reduce the incidence of AF in cancer patients.

## Data Availability Statement

The datasets presented in this study can be found in online repositories. The names of the repository/repositories and accession number(s) can be found in the article/Supplementary Material.

## Ethics Statement

The studies involving human participants were reviewed and approved by Medical Ethics Committee of East Hospital, Tongji University. The patients/participants provided their written informed consent to participate in this study.

## Author Contributions

CG, CW, and YZ designed the experiments and analyzed data. TY, SZ, and YS analyzed all the data, wrote the manuscript, and interpreted results. CX and MZ performed data acquisition and part of the data analysis. All authors reviewed the final manuscript and approved it for publication.

## Conflict of Interest

The authors declare that the research was conducted in the absence of any commercial or financial relationships that could be construed as a potential conflict of interest.

## Publisher's Note

All claims expressed in this article are solely those of the authors and do not necessarily represent those of their affiliated organizations, or those of the publisher, the editors and the reviewers. Any product that may be evaluated in this article, or claim that may be made by its manufacturer, is not guaranteed or endorsed by the publisher.
