# Supplementary material for: Pan-Cancer Analysis of Atrial-Fibrillation-Related Innate Immunity Gene ANXA4
Source: Front Cardiovasc Med. 2021 Sep 3;8:713983. doi: 10.3389/fcvm.2021.713983 (PMC8446278; doi:10.3389/fcvm.2021.713983)
Supplement: Supplementary file 1 [file Data_Sheet_1.docx]

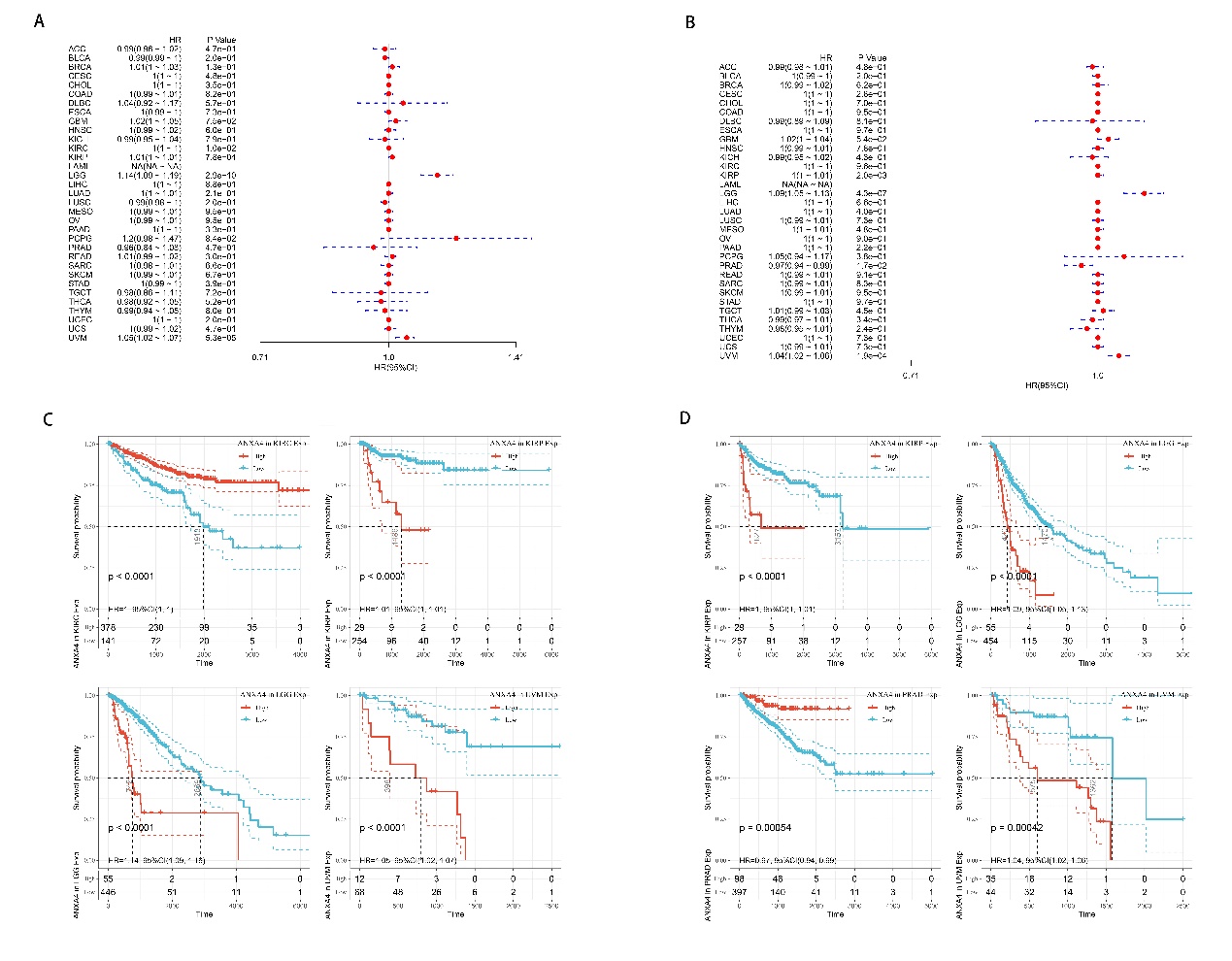


**Figure S1** Relationships between *ANXA4* expression levels and prognosis. **(A)** Forest plots of disease-specific survival (DSS). **(B)** Forest plots of progression-free interval (PFI). **(C)** Kaplan-Meier curves for DSS. **(D)** Kaplan-Meier curves for PFI.
